# Structural Behavior of a Composite Curtain Wall Fabricated by the Fused Deposition Modeling 3D Printing Method

**DOI:** 10.3390/polym14071431

**Published:** 2022-03-31

**Authors:** Min Jae Park, Jaehoon Bae, Young K. Ju

**Affiliations:** 1 School of Civil, Environmental and Architectural Engineering, Korea University, Seoul 02841, Korea; alswo8739@korea.ac.kr; 2 Department of Architectural Design, College of Engineering Science, Chonnam National University, Jeonnam 59626, Korea; skycity-bjh@jnu.ac.kr

**Keywords:** FDM 3D printer, ABS-M30, cement cladding, harmony search algorithm, FEM

## Abstract

In this era of the fourth industrial revolution, the integration of big data and 3D printing technology with the construction industry has maximized productivity. Currently, there is an active effort to research the optimal cladding structure through 3D printing technology to reduce production costs. This paper proposes a new type of 3D print curtain wall, using a high-strength ABS-M30 polymer panel, which is stronger than the standard acrylonitrile butadiene styrene (ABS) polymer, as an internally reinforced structure. This structure is fabricated via fused deposition modeling, a 3D printing method, to reduce the weight of the general cement panel. In addition, the shape of the polymer board was designed; three shapes were considered—O, W, and X types—which aided in further reducing the weight of the cladding. After comparing the center deformation of the structure through a lateral load test and finite element method analysis, the optimal model was selected. The measured data of the two methods at a design wind speed of 100% showed a difference of approximately 10%; however, at 150% of the design wind speed, the difference between the two sets of data increased to 27%.

## 1. Introduction

The technology of 3D printing (3DP), also known as additive manufacturing, is gradually dominating the global market owing to its design flexibility, mechanical automation, and minimal waste generation. In the era of big data, the application of information to the construction industry has become a global trend; this has also accelerated the integration of the construction industry and 3DP technology. In recent years, several specific houses have been printed using 3DP; for example, WinSun printed a house with an area of approximately 200 m^2^ in Shanghai, China. The cost was approximately USD 4800, which is lower than traditional construction costs [1,2,3]. Moreover, several companies and institutions are committed to manufacturing structural elements through 3DP technology. Among these, the multifunctional and acoustic damping wall elements established by the XtreeE team in 2015 were known as the largest 3D printed concrete elements available at that time [4]. In addition, according to the data compiled by Tay, Y. W. D. et al. [5], with the commencement of the fourth industrial revolution, research on 3DP technology for building and construction applications has increased exponentially. This reflects the comprehensive developments in the field of 3DP in many countries. Consequently, increasing numbers of research initiatives are being proposed.

Cladding, a part of the external protective structure of buildings, not only provides support for the exterior of the building but also prevents internal concrete and other materials from being damaged [6]. By comparing the energy properties of different cladding materials, Takano, A. et al. [7] recognized the brick cladding feature as the best material to replace wood planking; however, the manufacturing of bricks involves significant energy costs and results in an increase in environmental pollution. As one of the representative cladding materials of modern architecture, glass is mostly set up in high-rise buildings. Owing to the characteristics of glass materials, they are susceptible to dynamic loads, high strains, and shocks; moreover, their inherent characteristics at different extreme loads are also different, resulting in the requirement of specific responses under specific extreme-load conditions [6,8,9]. Alternatively, fiber-reinforced polymer (FRP) composite cladding systems are a potential candidate for use as cladding, considering their several advantages [10]. However, the performance of this system in terms of fire resistance is poor; moreover, when considering the emerging FRP composite system with bio-based composition, factors pertaining to heat, degradation, and other issues must be considered [11]. Concrete cladding is widely used in civil and public buildings, owing to its unique material properties that are recognized by the general public [6]. Kim, H. B. [12] proposed a new type of cement cladding composed of cement and polylactic acid (PLA) polymer. In this previous work, a lightweight and high-performance PLA polymer that is widely used in 3DP was selected as the main part of a multilayer reinforcement system, thereby reducing the overall weight of the cement cladding. However, this research was at the theoretical stage; moreover, verification was performed using only the finite element method (FEM) analysis, which reduces the reliability of the obtained results. Subsequently, a PLA polymer-based space truss structure was proposed by Bae, J. H. et al. [13]. However, owing to the small element size in the space truss, the production of space trusses is limited; moreover, there exists a paucity of research in this field, and additional models are required for further evaluations. Therefore, this study uses the fused deposition modeling (FDM) method to print a polymer reinforcement board, and further validation is performed through a lateral load test and FEM analysis.

Thermoplastic materials are commonly used in FEM analysis. Among them, PLA and acrylonitrile butadiene styrene (ABS) are widely used [14]. Shubham, P. et al. [15] demonstrated that, as the layer thickness decreases, the performance of the 3D printed ABS sample is improved. In addition, Tanoto, Y. Y. et al. compared the mechanical properties of PLA and ABS and determined that the tensile strength of ABS is significantly higher than that of PLA; however, its dimensional accuracy is lower than that of PLA [16]. In this research, tensile strength is prioritized over the accuracy of the sample, and the 3D printed curtain wall belongs to small parts; therefore, ABS was used as the polymer material in this research. This paper proposes a 3D printed composite curtain wall (3DP CCW) system that divides a general cement cladding panel into three equal parts; thereafter, a lightweight ABS polymer panel is used to replace the middle panel as the internally reinforced structure by employing 3DP technology. In addition, the shape optimization of the internal ABS polymer panel was performed to further reduce the overall cladding weight, while maintaining the stability of the structure. Structural deformation was then compared using FEM analysis and a lateral load test, and the optimal model was selected.

## 2. Materials and Methods

### 2.1. Fused Deposition Modeling 3D Printing Method

**Figure 1:**
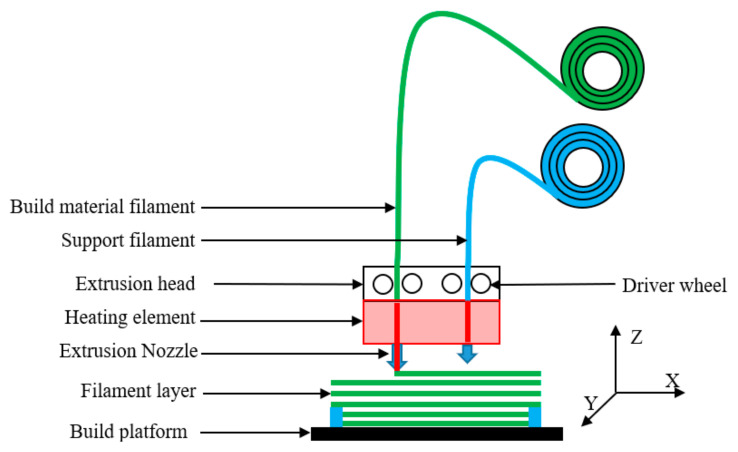
Working principle of fused deposition modeling.

FDM is one of the standard 3D printing methods; material extrusion (MEX) was developed by Stratasys Ltd. (Eden Prairie, MN, USA). A variety of materials can be used to fabricate components via FDM, including thermoplastic polymers, elastomers, and investment casting wax. FDM can directly generate 3D components from a computer-generated solid or indirectly create model data from a 3D object digitization system to create the required components. The working principle of MEX, generically known as FDM, is illustrated in Figure 1. A heated nozzle is controlled by a computer and it moves along the X-Y direction according to the profile information of the product; each layer is composed of a raster and contour. The raster angle ranges from 0 to 180° with respect to the *X*-axis, which is within the printing range along the direction specified by each layer. First, the support material filament is sent to the heating element, and the support is formed and attached to the building platform. Next, the thermoplastic material filament is sent to the heating element. The filament is heated and converted to a semi-liquid state, extruded through the extrusion nozzle, and, then, attached to the build platform according to the cross-sectional profile. After rapid cooling, a specific thick contour chosen in slicer software is formed; subsequently, the heated nozzle moves along the Z-direction to cycle to the contour of the next layer and form another part [17,18].

### 2.2. ABS-M30 Polymer

ABS is an amorphous polymer that has no fixed melting point. Consequently, it is a suitable material for injection molding under different conditions. In addition, four ABS derivatives have also received extensive attention with regard to MEX: ABSi, ABS-M30, ABS-ESD7 (electrostatic dissipative), and polycarbonate/ABS [19]. Among these, ABS-M30 is superior to ABS in terms of tensile strength, impact resistance, flexural strength, parts durability, and layer adhesion, making it an ideal choice for high-quality parts. Table 1 lists the primary material properties of ABS-M30, which is used to fabricate and analyze 3D printed polymer boards [20].

### 2.3. Cellulose-Fiber-Reinforced Cement Board

**Figure 2:**
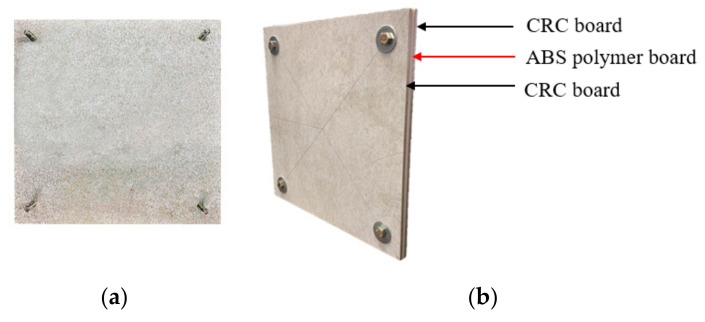
Details of the cellulose-fiber-reinforced cement board: (**a**) CRC board; (**b**) 3D printed composite curtain wall.

Fiber cement, an artificial building material, is created by mixing Portland cement, fiber, and water; the weight of the fiber is approximately 5–20% of the weight of cement. Accordingly, the continued addition of cellulosic fiber and silica sand after autoclaving results in the formation of a cellulose-fiber-reinforced cement (CRC) board. Owing to the addition of the cellulosic fiber, the overall mechanical strength and flexibility of the CRC board were significantly improved compared with the general cement board. Moreover, CRC boards have gradually become an ideal material for exterior cladding panels owing to their excellent fire resistance, durability, and easy installation [21]. In this study, CRC boards were used on both sides of the 3DP CCW, as shown in Figure 2.

### 2.4. Harmony Search Algorithm

The harmony search algorithm (HSA) is a meta-heuristic algorithm that was proposed by Geem and Kim in 2001 [22]; this algorithm was inspired by the process involved in a musical performance. Considering the harmonious analogy in music as a vector for optimization solutions, the improvisation analogy of the musician is used as a search solution in optimization technology. The impromptu performance of musicians determines the harmony of music through aesthetic standards, similar to the overall solution of the objective function in the optimization process. The objective function value is determined by the set of values assigned to each decision variable [22]. In this study, the HSA was used for finding the optimal design for the shape of polymer boards with minimum weight. The HSA optimization process first initializes the HSA parameters and then initializes the harmony memory; subsequently, it improvises a new harmony memory and updates the new memory. This process of improvising and updating is repeated until the conditions are satisfied. In an actual situation, HSA is completely controlled by three rules for improvising new memory: random selection, memory consideration, and pitch adjustment. The four important parameters in the HSA are as follows: harmony memory consideration rate (HMCR), harmony memory size (HMS), pitch adjustment rate (PAR), and bandwidth (bw) [23]. In this study, the HSA parameters were defined as HMCR = 0.8, HMS = 200, PAR = 0.2, and bw = 1.

## 3. 3D Printed Composite Curtain Wall (3DP CCW) System

### 3.1. Introduction

**Figure 3:**
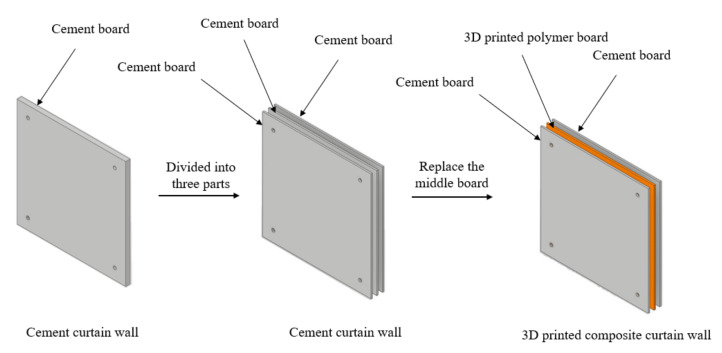
Concept of the 3D printed composite curtain wall (3DP CCW).

**Figure 4:**
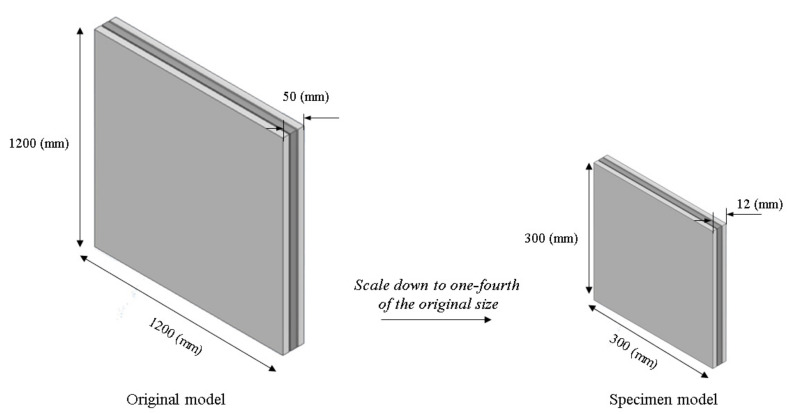
3DP CCW specimen model.

The 3DP CCW system is inspired by the advantages of sandwich cladding and FRP composite cladding, which guarantee high stiffness and strength, while reducing the overall weight [11]. The concept of 3DP CCW is to divide the general cement cladding into three equal parts, replace the middle cement board with a 3D printed polymer board, and then re-connect the three boards as a whole using bolts. Figure 3 illustrates the concept of the 3DP CCW. The size of the 3DP CCW was 1200 mm × 1200 mm × 50 mm (length × width × thickness). Since the printable size of the used FDM 3D printer is 400 mm × 355 mm × 406 mm (length × width × height), to print the entire model, the model must be divided into four parts and printed separately; these four parts would then need to be spliced together. However, directly using the splicing method causes the experimental results to be unpredictable; this incorporates additional variables in the experiment, making it difficult to determine the final result. Therefore, the 3DP CCW model was scaled down to one-fourth of its dimensions, and the test was conducted; moreover, after calculating the scale factor (S.F.), we combined the test data with the S.F. to obtain the deformation of the original 3DP CCW. The specific test specimen for the lateral load test is shown in Figure 4.

### 3.2. Shape Optimization of the 3DP CCW

**Figure 5:**
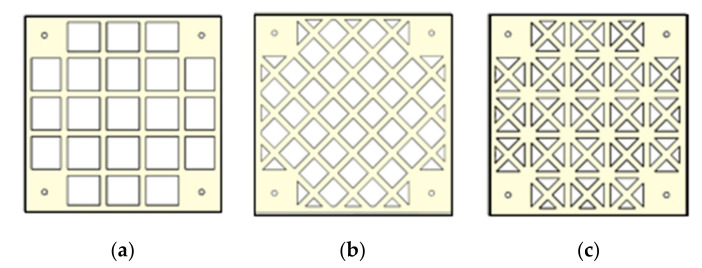
Shape of ABS polymer board: (**a**) O type; (**b**) W type; (**c**) X type.

In the 3DP CCW system, the ABS polymer board is the primary part of the overall structure and it contributes toward a lightweight structure. For further improvements, a necessary structural shape design was considered to further reduce the weight of the structure. The proposed three shapes of the ABS polymer board were considered from the previous study [24]: without reinforcement (O type), with reinforcement (W type), and with hybrid reinforcement (X type); these are illustrated in Figure 5. The O type has a simple rectangular grid texture, and the polymer is stacked in a manner similar to a metal wire mesh. In this case, the load was evenly dispersed through the grid lines and, then, transferred to the board at the rear. The W type refers to a concrete slab with yield-line patterns, subjected to a uniform load with sample support, which is a diagonal grid [25]. The polymer is stacked in a direction perpendicular to the stress contour; consequently, stress is effectively dispersed along this direction, which reduces the overall deformation to a certain extent [12]. Finally, the X type combines the aforementioned two types, thereby further improving stress dispersion and minimizing the overall deformation. Moreover, four-corner reinforcement was achieved by filling with the ABS polymer, and the distance for this reinforcement is 60 mm [26].

#### 3.2.1. Unit Size Optimization of O-Type ABS Polymer Board

**Figure 6:**
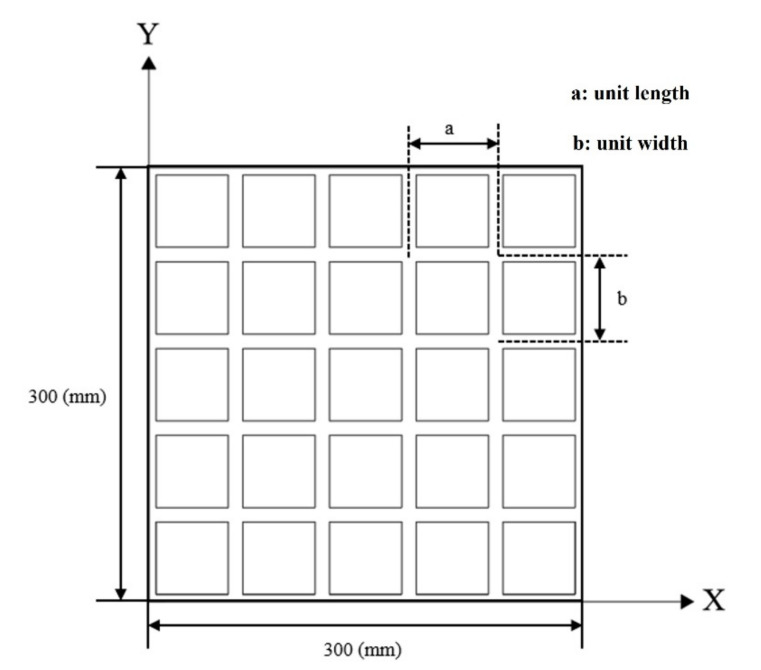
Optimization modeling of the O-type board.

The research goal of the 3DP CCW system was to reduce the overall weight, and the O-type board only corresponds to the unit shape of a polymer board. In addition, it is necessary to optimize the size of the rectangular grid inside the O-type board. For unit size optimization, the O-type board is analogous to grid floors, and the boundary condition is defined as simply supported on all edges under a uniformly distributed load. The load is related to a design wind speed in the lateral load test; the design wind speed was set as 10 m/s. The unit length (a) and width (b) in the rectangular grid are considered as the main parameters, and the distance between the two unit models was fixed at 10 mm; moreover, the thickness of the model remained unchanged at 4 mm. Based on Equations (1) and (2), the volume and center deflection object function for the O-type board can be calculated. HSA optimization analysis was performed using the MATLAB R2020 optimization toolbox. The optimization model for the O-type board is shown in Figure 6 [27]. The first constraint is the limitation of the unit length (a) and width (b). Owing to the four-corner reinforcement of the polymer board, the sizes of “a” and “b” are limited to 10–160 mm. Moreover, the deflection should be less than L/250, where L is the curtain wall length.

(1) $$V_{O}=360,000-\frac{302,400ab}{\left(a+10\right)\left(b+10\right)}$$

(2) $$D_{O}=\frac{960\times 10^{-6}}{\pi^{6}}\left(\frac{300^{4}\times 12\times \left(a+10\right)\times \left(b+10\right)}{EI_{x}\times a\left(b+10\right)+EI_{y}\times b\left(a+10\right)+0.312ab\left(a+10\right)\left(b+10\right)}\right)\leq \frac{L}{250}=1.2\;\mathrm{mm}$$

where *V_O_* is the Volume of the O-type board, *a* is the length, *b* is the width, *D_O_* is the deflection at the center of the O-type board, *E* is the modulus of elasticity for ABS polymers, *I_x_* is the moment of inertia about the *x*-axis, *I_y_* is the moment of inertia about the *y*-axis, and *L* is the curtain wall length.

**Figure 7:**
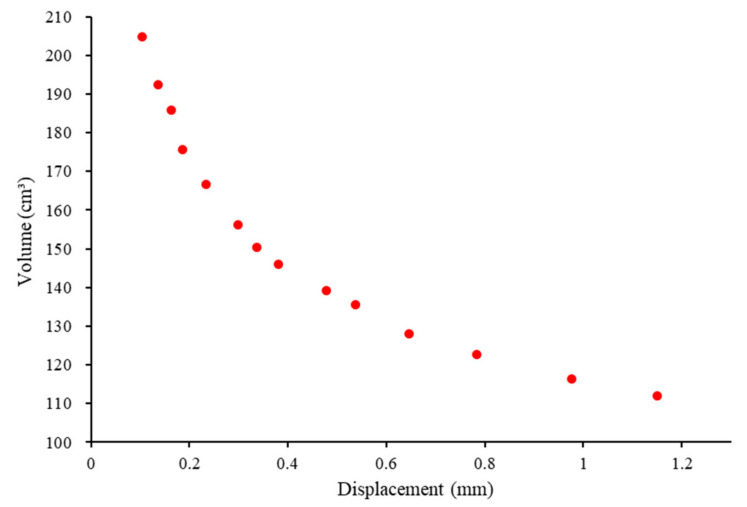
O-type optimization analysis results based on the HSA.

The results of the O-type optimization analysis are shown in Figure 7. After excluding the data that did not satisfy the constraints and sorting out the remaining data, 14 sets of values for the unit length and width that satisfied the requirements were obtained, as listed in Table 2. Considering that the four-corner reinforcement distance is 60 mm, a unit size that is too large or too small would cause printing difficulties or uneven force distribution; therefore, the unit length and width should be equal to the corner reinforcement distance, which is 60 mm. Thus, data group 7 shown in Table 2 was chosen to fabricate the O-type board.

#### 3.2.2. Unit Size Optimization of W-Type ABS Polymer Board

**Figure 8:**
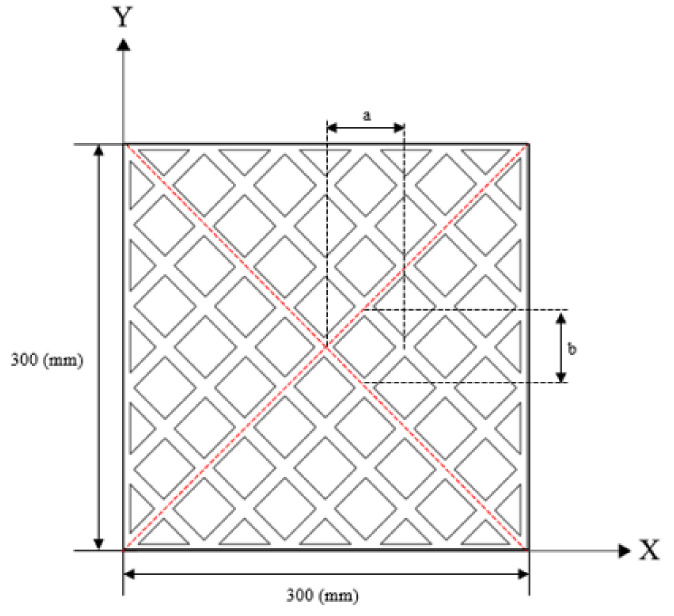
Optimization modeling of the W-type board. a: unit length; b: unit width.

The unit size optimization process for the W-type polymer board is similar to that for the O-type board, where the boundary conditions and load remain fixed, as shown in Figure 8. Owing to the change in the internal cross-sectional area of the board, optimization analysis was conducted using Equations (3) and (4). To determine the minimum deflection, the moment of inertia in the optimization formula is selected as the cross-section at the intersection of two unit models, and the constraints are the same as those for the O-type board.

(3) $$V_{W}=360,000-\frac{360,000\times ab}{\left(a+14\right)\left(b+14\right)}$$

(4) $$D_{W}=\frac{960\times 10^{-6}}{\pi^{6}}\left(\frac{300^{4}\times 12\times \left(a+14\right)\times \left(b+14\right)}{EI_{x}\times a\left(b+14\right)+EI_{y}\times b\left(a+14\right)+0.312ab\left(a+14\right)\left(b+14\right)}\right)\leq \frac{L}{250}=1.2\;\mathrm{mm}$$

where *V_W_* is the Volume of the W-type board, *a* is the length, *b* is the width, *D_W_* is the deflection at the center of the W-type board, *E* is the modulus of elasticity for ABS polymers, *I_x_* is the moment of inertia about the *x*-axis, *I_y_* is the moment of inertia about the *y*-axis, and *L* is the curtain wall length.

**Figure 9:**
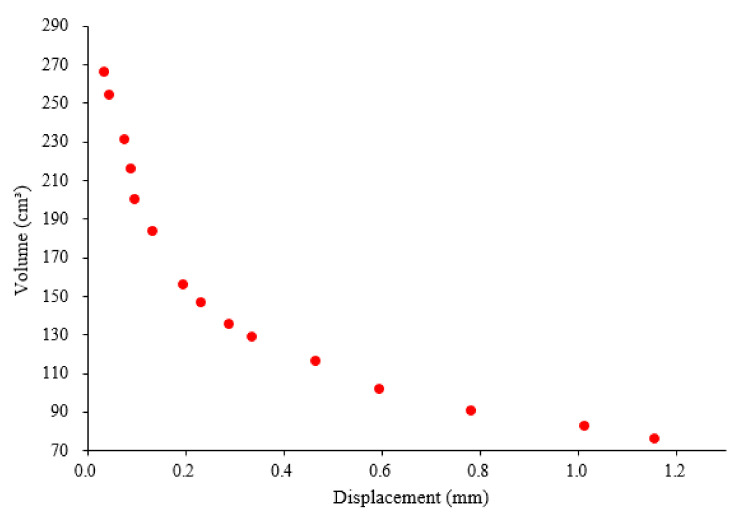
W-type optimization analysis results based on the HSA.

The HSA analysis results for the W-type board are shown in Figure 9. After deleting and sorting out the data, 15 sets of unit sizes that satisfied the requirements were obtained, as listed in Table 3. The unit length and width were chosen to be approximately equal to the four-corner reinforcement distance; therefore, data group 8 was selected to fabricate the W-type model. Furthermore, the X-type board was fabricated by combining the W- and O-type boards obtained after optimization.

## 4. Lateral Load Test and FEM Analysis for 3DP CCW

### 4.1. Laster Load Test

#### 4.1.1. Test Plan

**Figure 10:**
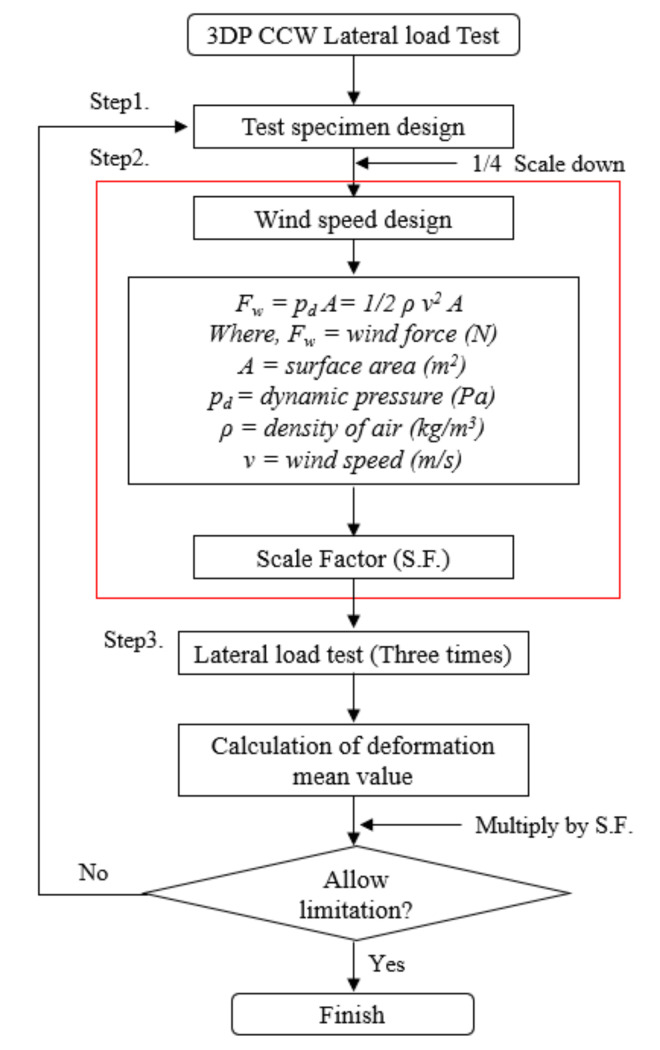
Process for the 3DP CCW lateral load test.

**Figure 11:**
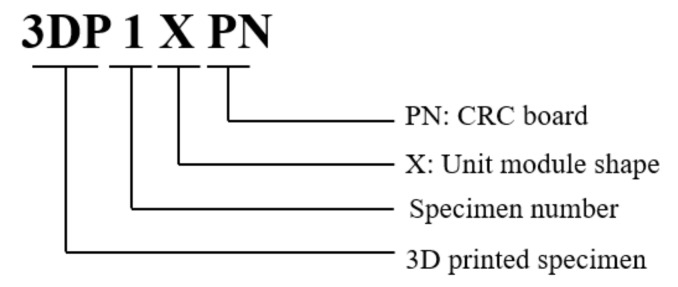
Definition of test specimen designation.

The 3DP CCW is a new type of cladding system, which must be subjected to a lateral load test to determine whether the deformation of the structural material satisfies the construction codes of precast concrete curtain walls [28]. In the lateral load test, after the test specimen is fixed in the wind tunnel laboratory, the wind speed is designed, and the wind pressure acting on the surface of the specimen is defined as the lateral load. Thereafter, using a laser distance sensor, the deformation of each specimen is measured. The specific process for the lateral load test is illustrated in Figure 10. The definition of the test specimen designations is shown in Figure 11. Including a standard specimen, a total of four test specimens were designed. In the 3DP CCW system, three different specimens were fabricated according to the three optimized ABS polymer board shapes. For the comparison experiments, a CRC cladding specimen without a polymer board replacement was used as the standard specimen, which was defined as PN. Details of these specimens are listed in Table 4.

#### 4.1.2. Wind Speed Design

In this test, the average wind speed in the Seoul area was selected as the design wind speed to calculate the wind pressure; the average wind speed was set at 26 m/s [29]. The wind pressure can be calculated from wind speed (Equations (5) and (6)). Since the wind speed of the wind tunnel equipment is limited to 20 m/s, by consulting the curtain wall design code, it can be observed that when 150% of the design wind speed was applied, the residual deformation was limited to 2L/1000. Therefore, the design wind speed in the lateral load test was 10 m/s, and the S.F. was confirmed after calculating the wind pressure of the original area; moreover, the parameters of the lateral load test were obtained based on Equations (5)–(7).

(5) $$P_{f,design}=\frac{1}{2}\rho V_{design}^{2}=405.6\;\mathrm{Pa}$$

(6) $$P_{f,test}=\frac{1}{2}\rho V_{test}^{2}=60\;\mathrm{Pa}$$

(7) $$S.F.=\frac{P_{f,design}}{P_{f,test}}\times \left(1+\alpha \right)=\frac{405.6}{60}\times \left(1+0.15\right)=8$$

where *P_f_*_,_*_design_* is the lateral load by design code, *ρ* is the density of air (1.2 kg/m^3^), *V_design_* is the wind speed by design code (26 m/s), *P_f_*_,_*_test_* is the lateral load in the later load test, *V_test_* is the wind speed in the lateral load test (10 m/s), and *α* is the safety rate (15%).

After calculating the wind pressure of the original area and the lateral load test using Equations (5) and (6), the S.F. calculated according to Equation (7) was 6.76; however, certain unpredictable factors in the test process may have caused the evaluated result to be significantly small; therefore, the safety rate was set to 15%, and the S.F. was set to 8. In the curtain wall design code, for the cladding structure test, the design wind speed was increased step-by-step to 150% of the design wind speed (the increase was achieved in three steps of 50%, 100%, and 150%), and at every step, the wind speed was retained for 10 s. The displacement of the structural material was measured to determine whether it satisfied the design criteria for material deformation, i.e., a value less than L/360. In addition, for 150% of the design wind speed, the displacement was measured after removing the wind to measure the residual deformation, and the allowable value should be less than or equal to 2L/1000. Therefore, two types of loading protocols were designed for the test. Among them, loading protocols #1 (L1) and #2 (L2) are 50% and 100%, and 50%, 100%, and 150% of the design wind speed, respectively. Since the design wind speed of each stage was maintained for 10 s, the accuracy of the data in the test was taken as the average value after three tests for each loading protocol. After determining the S.F., we multiplied the test result by the S.F. to obtain the deformation of the original model. Similarly, for 150% of the design wind speed, the residual deformation of L2 was calculated based on 2L/1000. The details of each loading protocol are listed in Table 5 and Table 6 [28].

#### 4.1.3. Test Setup

**Figure 12:**
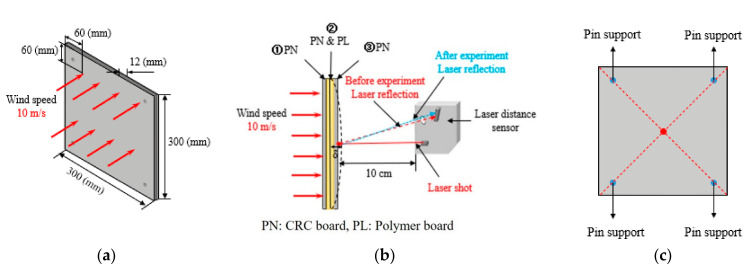
Details of the test specimen setting: (**a**) front view; (**b**) side view; (**c**) rear view.

Before the test, the setting of the specimens and selection of the displacement measuring equipment must be considered. Consequently, it is necessary to design a set model for the test specimen and use it as the basis for the test. Moreover, as the displacement of the specimens was small, a laser distance sensor with higher accuracy was selected to measure the displacement of each specimen. Since the measurement displacement range of the laser distance sensor was 30–130 mm, the laser sensor was placed at the center, at a distance of 10 cm from the back of the specimen for measurement. The details of the test specimen setting model are shown in Figure 12.

**Figure 13:**
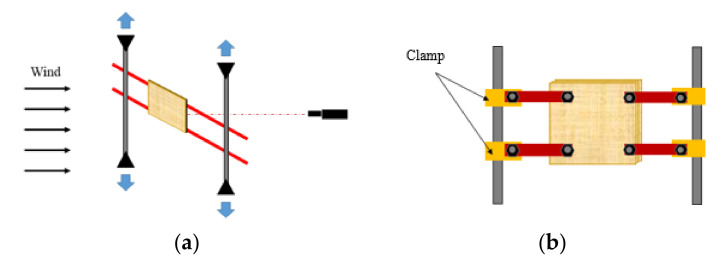
Specimen setup details: (**a**) vertical member; (**b**) horizontal member.

In addition, the height of the wind tunnel laboratory was 2 m; therefore, the vertical and horizontal members were required to fix the specimen, as shown in Figure 13. In the wind tunnel laboratory, the vertical members were compressed and fixed to the ceiling and floor; the horizontal members were clamped to the vertical members of the specimen.

**Figure 14:**
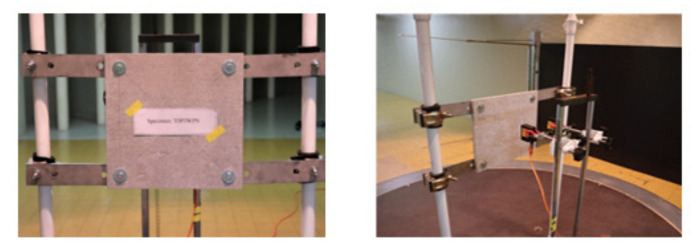
Installation of the test specimen.

All components were installed in the wind tunnel laboratory, and the laser distance sensor was fixed and accurately set up behind the specimen. The specific installation process of the specimen is shown in Figure 14. After all the preparations were completed, the lateral load test was performed according to the loading protocol.

### 4.2. FEM Analysis

**Figure 15:**
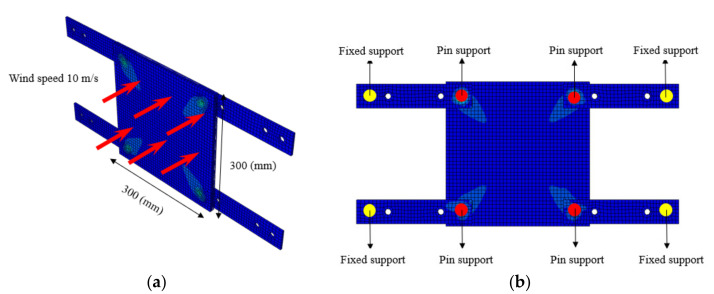
Finite element modeling: (**a**) front view; (**b**) rear view.

The structural analysis of the 3DP CCW was conducted using commercial software, Abaqus/CAE 2017. The analysis model of the 3DP CCW was regarded as an eight-node solid element. The finite element model was modeled as same as the specimen (three layers), and the contact surfaces was tied because the loads were not huge. The connection between the specimen and the vertical member in the lateral load test was set to pin support, and the connection between the vertical and horizontal members was regarded as fixed support, as shown in Figure 15. In the lateral load test recorded the time taken when the wind speed changed from 0–50% to 50–100% during the process where the wind speed increases; subsequently, the wind speed was accelerated through a similar time interval in the FEM analysis. Moreover, L1 and L2 were performed in the same manner as the lateral load test. Further, in the FEM analysis, the attachment method considered the surface-to-surface attachment of the CRC board and ABS polymer board [12,13].

## 5. Results and Discussion

### 5.1. Laster Load Test and FEM Analysis Results

#### 5.1.1. Lateral Load Test Results

**Figure 16:**
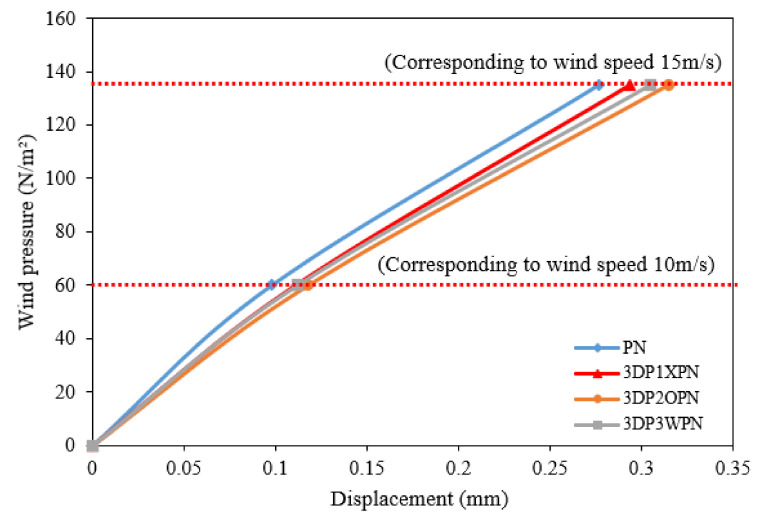
Wind pressure–displacement diagram of the lateral load test.

**Figure 17:**
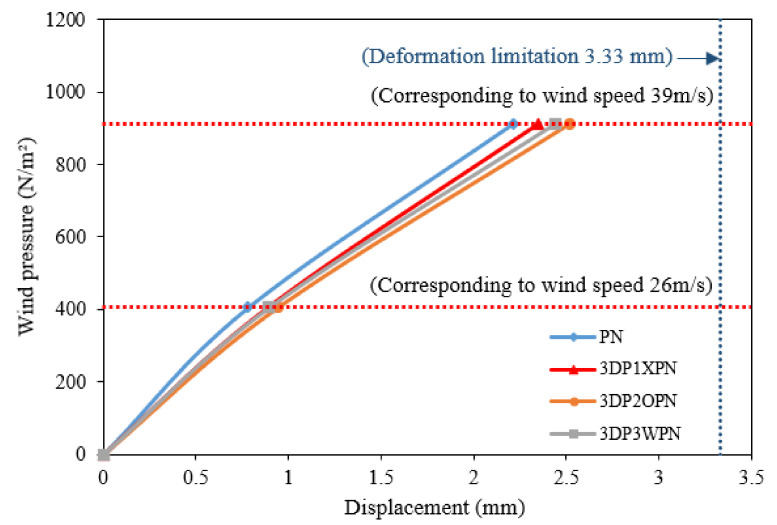
Wind pressure–displacement diagram of the model in the original area.

According to L1 and L2, three experiments were performed for each specimen. When collating the data, the maximum displacement values in the two sets of loading protocols were measured; the maximum displacement was obtained when the original area wind speed acted upon the 3DP CCW system according to the S.F. The test results obtained are listed in Table 7. The corresponding wind pressure caused by the design wind speed was calculated using Equation (6). The maximum displacement values of L1 and L2 at 100% and 150% of the wind speed were obtained, as shown in Figure 16. In addition, according to the cladding or curtain wall code, the allowable range in structural deformation of the cladding is L/360 (3.33 mm) under wind pressure. By multiplying the experimental data with the S.F., it was determined that all specimens were within the allowable range of structural deformation. When the wind speed was reduced by 150% of the design wind speed to 0, the residual deformation of the structure was also within the allowable range of 2L/1000 (2.4 mm). Figure 17 shows the wind pressure–displacement diagram of the 3DP CCW model in the original area.

#### 5.1.2. FEM Analysis Results

The results of the FEM analysis are listed in Table 8. It can be noted that the deformation of the structure is consistent with the prediction, and the maximum deformation of the structure occurs at the center. The FEM analysis results can only determine the absolute deformation of the structure, whereas the deformation of the lateral load test measures the relative deformation. Therefore, the deformation at the edge of the board was subtracted from the deformation at the center of the structure, similar to the deformation of the structure in the FEM analysis, which is listed in Table 9.

### 5.2. Discussion

**Figure 18:**
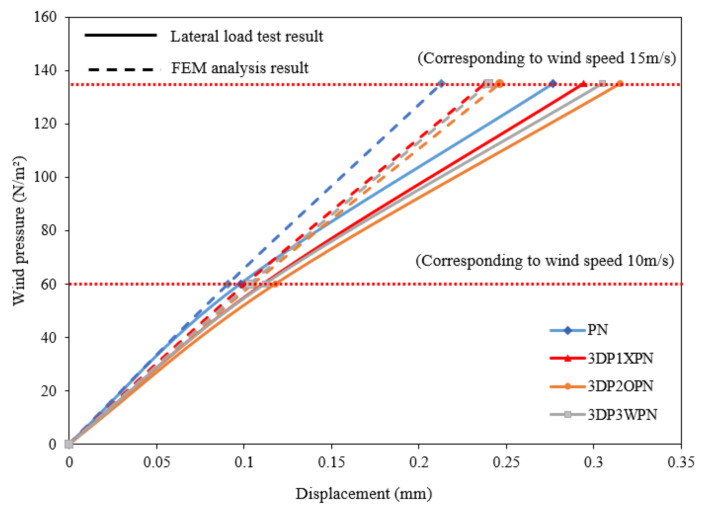
Wind pressure–displacement diagram of FEM analysis and lateral load test.

Figure 18 shows the FEM analysis and test results. When 100% of the design wind speed impacts the 3DP CCW specimens, the average difference between the two sets of data is approximately 10%. However, as the wind speed increased from 100% to 150% of the design wind speed, the difference between the two sets of data gradually increased to 20%, as shown in Table 10.

In the L1 results listed in Table 10, the lateral load test and FEM analysis results have similar trends, but the difference between the two sets of data of the 3DP2OPN specimen is 12.4%; this could be because the design process of the O-type model forms a weak specimen that is supposed to obtain the largest deformation in the test. The larger deformation during the test may have resulted in a higher impact on the reinforcement bolts at the four corners than the other test specimens. In addition, as the wind speed increases, the position of the laser displacement sensor and the rotation of the connection will gradually increase in the test, making the test result of all specimens higher than that of the FEM analysis, which leads to the L2 result, where the magnitude of the difference increased to 20%.

The polymer reinforced 3D printed claddings, printing methods, materials, internal reinforcement structures, and minimum deformations are listed in Table 11. According to the list, the maximum central deformation results of the original 3DP CCW model are similar to the results of the previous two studies; however, further research is required to make the results more accurate.

**Figure 19:**
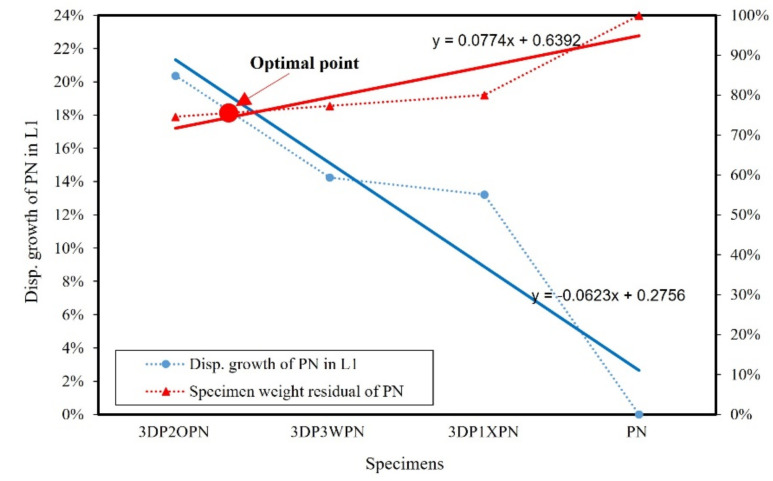
Optimal point of the later load test in loading protocol #1 (L1).

**Figure 20:**
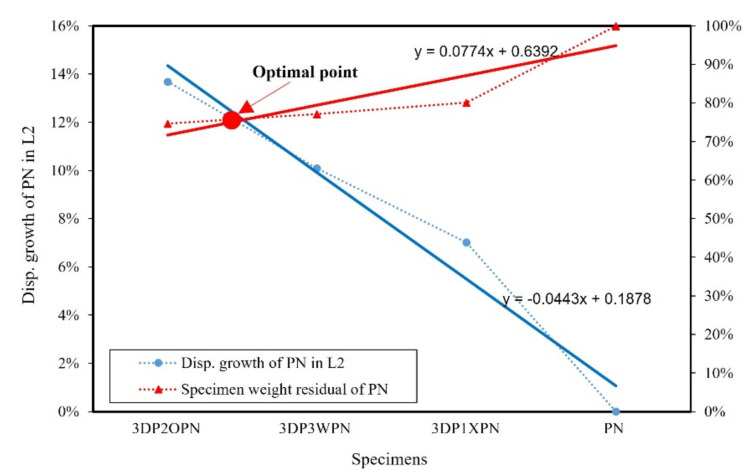
Optimal point of the later load test in loading protocol #2 (L2).

**Figure 21:**
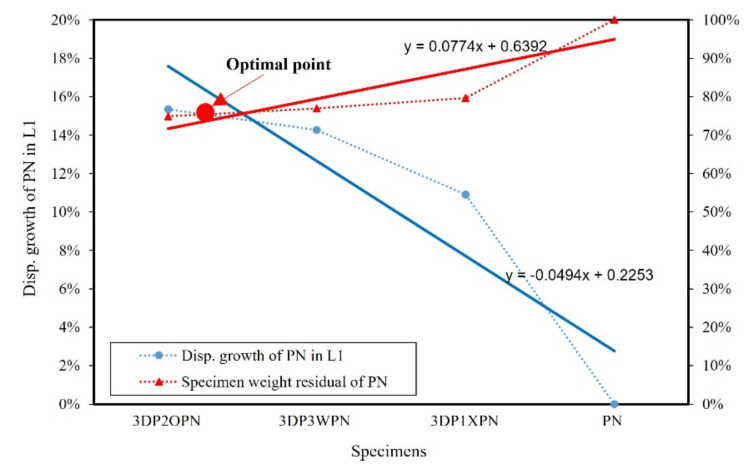
Optimal point of the FEM analysis in loading protocol #1 (L1).

**Figure 22:**
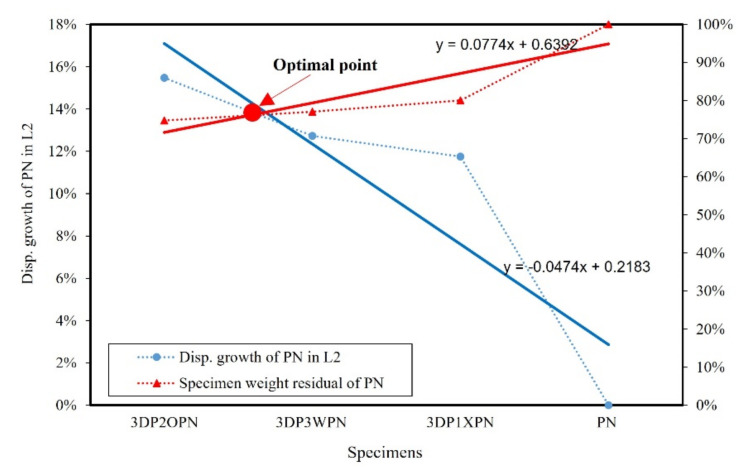
Optimal point of the FEM analysis in loading protocol #2 (L2).

Let us now consider the selection of the optimal shape of the 3DP CCW specimen. The result of specimen PN was considered as the standard value. The weight of each specimen was divided by the weight of PN, which was defined as the specimen weight residual of PN. Furthermore, the precast concrete curtain wall code [28] stipulated that the deformation and residual deformation under 100% and 150% of the design wind speed had to be recorded. Thus, the increase in the displacement of the 3DP CCW specimen, when compared to the PN displacement, was recorded as the displacement growth rate in L1 and L2. The optimal points of the lateral load test and FEM analysis in L1 and L2 are shown in Figure 19, Figure 20, Figure 21 and Figure 22. The trend function was used to observe the results clearly.

According to Figure 19, Figure 20, Figure 21 and Figure 22, irrespective of the optimal point in the lateral load test (L1 or L2), it can be observed that the optimal specimen of 3DP CCW is 3DP2OPN. In contrast, in the case of L1 in the FEM analysis, the optimal point was closer to that of the 3DP2OPN specimen, and, in the case of L2, the optimal point was closer to that of 3DP3WPN; this could be because of the influence of the pin connection increases with the increase in wind speed, causing the difference between the test and FEM analysis data to reach 27%, which affects the optimal point position.

## 6. Conclusions

The development of 3DP technology in the construction industry has prompted the development of several new cladding materials to achieve a freeform structure. Among them, cement cladding is a widely used exterior cladding that has limitations with respect to the formwork and higher weight, which inevitably increases the construction budget. However, in this study, the FDM 3DP method was used to fabricate a lightweight ABS polymer board as the internal reinforcement structure to achieve a 3DP CCW. Moreover, after optimizing the design of the ABS polymer board, a lateral load test and FEM analysis were performed; then, the 3DPCCW deformation was evaluated and the optimal model was selected. The FDM method is an additive manufacturing technology that was used in this study to produce an internal reinforcement structure, in which the ABS-M30 material was selected owing to its higher tensile and flexural strengths. Moreover, 3DP CCW was obtained by replacing the ABS-M30 polymer board in the central panel, which divided the general cement cladding panel into three equal parts. The size of the 3DPCCW was 1200 mm (L) × 1200 mm (W) × 50 mm (T), and because of the size limitation of the FDM machine, the 3DP CCW model was scaled down to one-fourth of the original size. Furthermore, three basic shapes were proposed to optimize the ABS polymer board: O, W, and X types. Subsequently, the unit size optimization of each ABS polymer board shape was performed. The unit size was determined to be 60 mm (L) × 60 mm (W) × 4 mm (T). The conclusions of this study are as follows:

(1) The lateral load test and FEM analysis were performed to measure the structural and residual deformations under wind speeds of 100% and 150% of the design wind speed; subsequently, the original model deformation under the average wind speed in the area of Seoul was calculated through the S.F. The FEM analysis and lateral load test results were compared, which were based on the premise that PN is used as the standard specimen. In terms of central deformation, the minimum deformation was obtained by the two methods for 3DP1XPN, and the difference between the data obtained by the two methods was 10% at 100% of the design wind speed. As the wind speed increased to 150% of the design wind speed, the data difference between the two methods increased to 23%. However, when considering the cost and deformation simultaneously, since the deformation of all the specimens and the residual deformation satisfied the requirements of the standard, the specimen with the smallest weight, i.e., 3DP2OPN, was selected as the optimal model.
(2) In the lateral load test, the cement board material cannot be guaranteed to be homogeneous. In addition, the pin connection will cause problems, such as rotation with the increase in wind speed, resulting in stress concentration at the connection. The strength of the bolts cannot be guaranteed to be consistent during the test, which could cause an uneven force on the cladding, thus making the result unreliable. Therefore, further studies are required to avoid these errors.
(3) Future research should be conducted with high-capacity test equipment to apply 26 m/s wind speed. In addition, the various connection details and full-scale tests should be conducted to investigate the actual behavior of the full-scale 3DP CCW and install it at the buildings.

## Figures and Tables

**Table 1:** Mechanical properties of ABS-M30.

Density (kg/m^3^)	Tensile Strength (MPa)	Tensile Elongation (%)	Modulus of Elasticity (MPa)	Flexural Strength (MPa)	Poisson’s Ratio	Polymer Type
1010	31.0	7.00	2230	60.0	0.2	Amorphous

**Table 2:** Numerical results based on the HAS for the O-type board.

Data Group	Length (mm)	Width (mm)	Volume (cm^3^)	Displacement (mm)
1	21	32	204.7	0.1041
2	23	39	192.5	0.1355
3	23	48	185.8	0.1631
4	33	39	175.6	0.1853
5	33	50	166.8	0.2329
6	56	39	156.2	0.2979
7	51	49	150.3	0.3374
8	58	49	146.0	0.3805
9	76	48	139.2	0.4786
10	50	82	135.6	0.5370
11	76	66	128.0	0.6464
12	67	92	122.8	0.7840
13	94	82	116.3	0.9758
14	94	98	112.1	1.150

**Table 3:** Numerical results based on the HAS for the W-type board.

Data Group	Length (mm)	Width (mm)	Volume (cm^3^)	Displacement (mm)
1	12	18	266.6	0.03409
2	12	24	254.7	0.04294
3	12	48	231.2	0.07562
4	44	16	216.2	0.08666
5	33	24	200.7	0.09554
6	24	47	184.1	0.1314
7	43	41	156.6	0.1936
8	45	48	147.0	0.2294
9	44	63	136.0	0.2876
10	72	46	129.0	0.3337
11	100	47	116.6	0.4626
12	99	63	102.1	0.5940
13	72	116	91.16	0.7806
14	144	76	82.77	1.012
15	134	95	76.10	1.155

**Table 4:** Details of the test specimen.

Specimen	Composition	Weight (kg)	Weight Residual of PN
PN	CRC panel	1.847	100%
3DP1XPN	CRC panel + X-type board	1.485	80.4%
3DP2OPN	CRC panel + O-type board	1.388	75.2%
3DP3WPN	CRC panel + W-type board	1.432	77.5%

**Table 5:** Loading protocol #1 (L1).

Time (s)	Wind Speed (m/s)	Wind Pressure (Pa)	Allowable Value
10	5 (50%)	15	
10	10 (100%)	60	L/360

**Table 6:** Loading protocol #2 (L2).

Time (s)	Wind Speed (m/s)	Wind Pressure (Pa)	Allowable Value
10	5 (50%)	15	
10	10 (100%)	60	L/360
10	15 (150%)	135	2L/1000

**Table 7:** Lateral load test results.

Specimen	Max. Disp. in L1 (mm)	Multiple by S.F. (mm)	Max. Disp. in L2 (mm)	Multiple by S.F. (mm)
PN	0.098	0.784	0.277	2.22
3DP1XPN	0.111	0.888	0.294	2.35
3DP20PN	0.118	0.944	0.315	2.52
3DP3WPN	0.113	0.904	0.305	2.44

**Table 8:** FEM analysis results.

Model Name	3DP CCW Maximum Deformation	ABS Polymer Maximum Deformation
PN	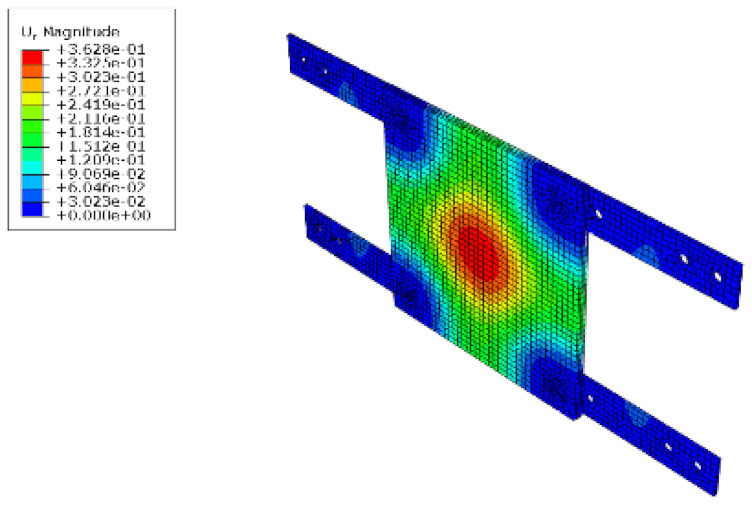	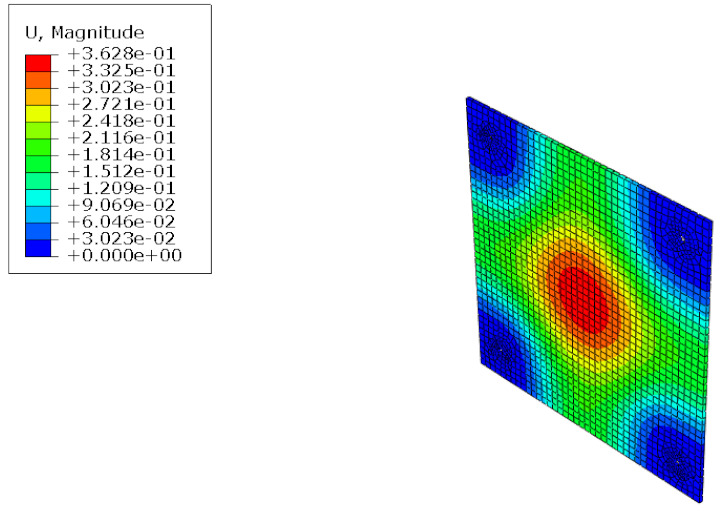
3DP1XPN	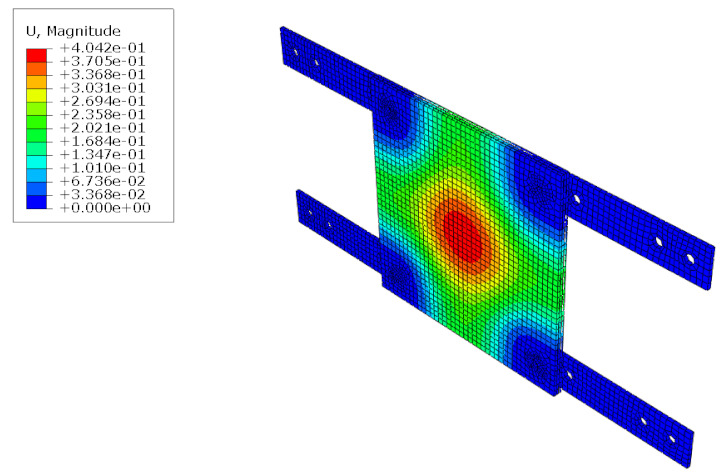	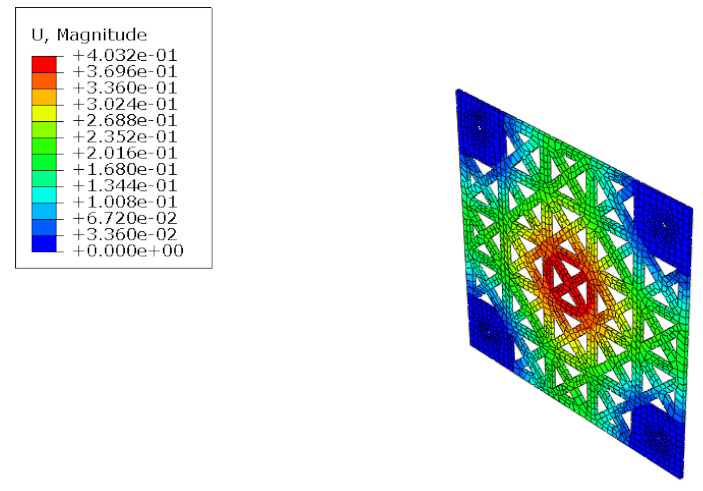
3DP20PN	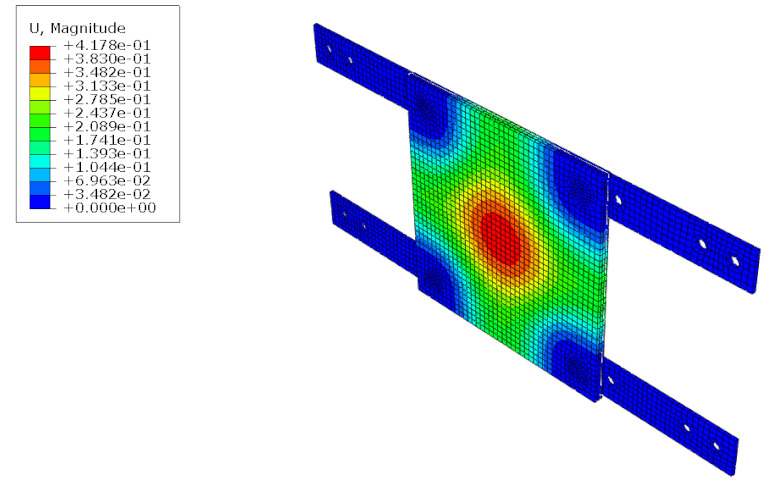	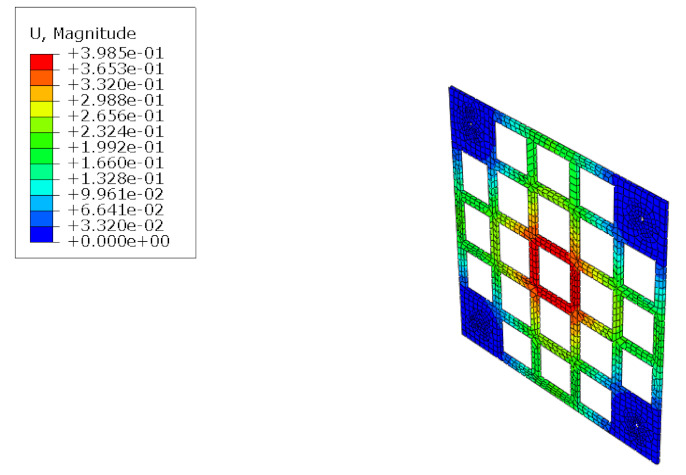
3DP3WPN	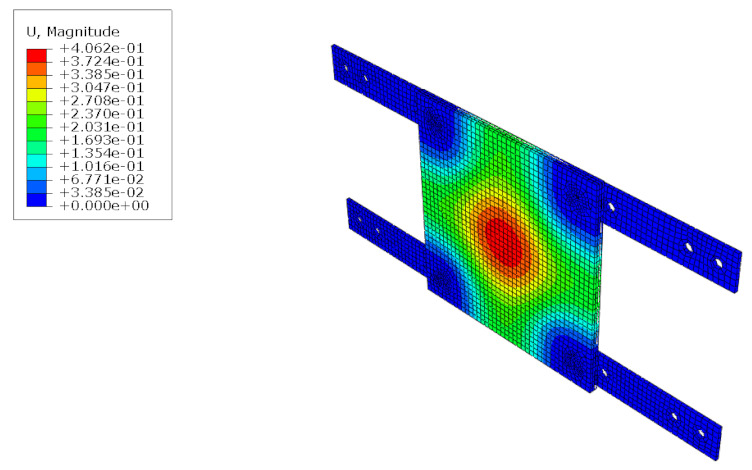	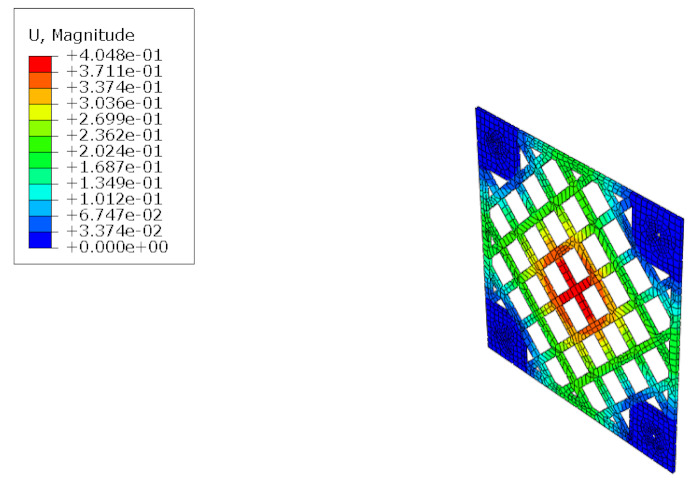

**Table 9:** Deformations by FEM analysis results.

Specimen	Max. Disp. in L1 (mm)	Multiple by S.F. (mm)	Max. Disp. in L2 (mm)	Multiple by S.F. (mm)
PN	0.091	0.728	0.223	1.78
3DP1XPN	0.101	0.808	0.238	1.90
3DP20PN	0.105	0.84	0.248	1.98
3DP3WPN	0.103	0.824	0.242	1.94

**Table 10:** Differences between FEM analysis and lateral load test results.

Specimen	PN	3DP1XPN	3DP2OPN	3DP3WPN
FEM analysis results in L1 (mm)	0.091	0.101	0.105	0.104
Test results in L1 (mm)	0.098	0.111	0.118	0.113
Differences	7.7%	9.9%	12.4%	8.7%
FEM analysis results in L2 (mm)	0.223	0.238	0.248	0.242
Test results in L2 (mm)	0.277	0.294	0.315	0.305
Differences	24.2%	23.5%	27.0%	26.0%

**Table 11:** List of 3D printed cladding methods and minimum deformation.

Reference	Method	Material	Internal Reinforcement	Minimum Deformation
[12]: Numerical investigation	3D concrete printing	PLA	Wire mesh (rectangular and diagonal)	R: 1.10 mm D: 1.11 mm
[13]: Numerical investigation	3D printing robot	PLA	Space truss	1.92 mm
[30]: Experimental and numerical investigation	FDM	PLA	Honeycomb structure	39.1 mm

## Data Availability

Not applicable.
